# Change in coronary heart disease hospitalization after chronic disease management: a programme policy in China

**DOI:** 10.1093/heapol/czac101

**Published:** 2022-11-24

**Authors:** Jingmin Zhu, Wei Wang, Jun Wang, Liang Zhu

**Affiliations:** Department of Epidemiology and Public Health, University College London, 1-19 Torrington Place, London WC1E 7HB, United Kingdom; Department of Social and Preventive Medicine, University of Malaya, Level 5, Block I, Kuala Lumpur 50603, Malaysia; Center for Health Policy Research and Evaluation, School of Public Administration and Policy, Renmin University of China, No. 59 Zhongguancun Street, Haidian District, Beijing 100872, China; Henan Province Yongcheng Central Hospital, Zhongyuan Road, Yongcheng, Shangqiu 476610, China

**Keywords:** Chronic disease management, coronary heart disease, hospitalization, direct medical cost, length of stay

## Abstract

This study aims at examining changes in coronary heart disease (CHD) hospitalization associated with a novel county-scale chronic disease management (CDM) programme policy implemented in March 2019 in China during the 13th Five-Year period (2016–2020). The CDM programme was designed to improve the health of populations with chronic diseases by means of an integrated way involving both county-level public hospitals and primary care institutes. Data originated from the medical files of CHD inpatients discharged from a secondary hospital from January 2017 to December 2020. A total of 6111 CHD patient records were collected. Univariate and multivariate regression analyses were performed to assess changes in hospitalization direct medical costs and length of stay of CHD patients. The mean direct medical cost of CHD hospitalization was 8419.73 Yuan, and the mean length of stay was 7.57 days. Results suggested that the implementation of CDM reduced hospitalization direct medical cost and bed days by about 23% (1956.12 Yuan at means) and 11.5% (almost 1 day at means), respectively. In addition, a further decreasing trend in medical costs over time was associated with chronic disease management. It is implied that chronic disease management is an effective way of relieving the medical and financial burden of hospitalization.

**Key messages**

- A national strategy for preventing and managing chronic diseases was launched in China in 2017. Chronic disease management services were designed and gradually provided for NCD patients nationwide.
- In a secondary public hospital located in Central China, the mean direct medical cost of CHD hospitalization was 8419.73 Yuan and the mean length of stay was 7.57 days.
- The implementation of the chronic disease management programme reduced direct medical costs and bed days of CHD hospitalization by about 23% and 11.5%, respectively.

## Introduction

The prevalence of non-communicable chronic diseases (NCDs) has become a public health challenge (Yang *et al.*, 2020). As one major NCD, coronary heart disease (CHD) has been a leading cause of death worldwide since 2016 (WHO, 2019). In addition, the economic burden of CHD is considerable, especially in low- and middle-income countries (Luengo-Fernández *et al.*, 2006; Abegunde *et al.*, 2007). Of all the new CHD deaths from 1990 to 2017 in the world, China accounted for 38.2%, the highest rate among all countries (Dai *et al.*, 2022). In 2019, 11 million patients were suffering from CHD in China (The Writing Committee of the Report on Cardiovascular Health and Diseases in China, 2020). In China, CHD is expected to be associated with a $494 billion (2010 USD) disease burden during 2012–2030 (Bloom *et al.*, 2018), a large part of which is the direct medical costs of inpatients (Chang *et al.*, 2012; Darba *et al.*, 2020).

Faced with an increasing prevalence of chronic conditions, the Chinese government launched a national strategy for preventing and managing chronic diseases in 2017, namely China’s Medium-to-Long Term Plan for the Prevention and Treatment of Chronic Diseases (2017–2025). Various actions have been taken such as promoting a healthy lifestyle, health status monitoring and improving the quality of health-care services (Kong, 2017). In particular, in order to promote population health and relieve the financial burden of NCDs, a novel county-scale chronic disease management (CDM) programme is designed to manage the health of populations with hypertension, diabetes, chronic obstructive pulmonary disease, stroke or CHD, which are associated with the highest mortality rates and disease burden in China. Without the programme, only primary care institutes were responsible for managing the health of populations with NCDs by means of basic health monitoring and medication. However, the CDM programme established an integrated way of health management based on an integrated health-care system with more medical resources and more advanced medical technology. It engages both county-level public hospitals and primary care institutes in predicting potential NCD patients, detecting NCD patients, implementing health-care interventions and providing health-care services for NCD patients. Four years after the establishment of the strategy, it is time to evaluate the outcome of the CDM programme in terms of reducing the financial burden of chronic conditions, which is the main objective of this study. Considering the high incidence of CHD and the heavy financial burden associated with CHD among all chronic diseases, this study takes CHD as an example and investigates how CHD hospitalization associated direct medical costs and length of stay change after the implementation of CDM.

In the literature, CDM is reported to be effective in reducing inpatient admissions, length of stay and medical costs of chronic conditions (Wagner, 1998; McAlister *et al.*, 2004; Brady *et al.*, 2013; Hamar *et al.*, 2015; Newman *et al.*, 2020). However, no one has examined the influence of chronic disease management on hospitalization and associated medical costs in China. In this study, we first attempt to inspect the changes in CHD hospitalization rate, direct medical cost and bed days of hospitalization in the context of China’s recent chronic disease management efforts.

Compared with existing literature, especially the studies on China, this study has several significant contributions. First of all, to the best of our knowledge, this study is the first attempt to evaluate the outcome of China’s chronic disease management from the perspective of chronic disease hospitalization and associated medical costs. Second, using a novel dataset from a secondary public hospital in China, we provide more up-to-date information on the direct hospitalization medical costs and length of stay of CHD in secondary hospitals as opposed to tertiary or rural township hospitals, over a longer period (Le *et al.*, 2015; Ding *et al.*, 2017; Wang *et al.*, 2019; Ramadhani *et al.*, 2020). Third, numerous studies have provided evidence on the influencing factors of the medical cost or length of stay of CHD (Currie *et al.*, 1997; Laurenti *et al.*, 2000; Bramkamp *et al.*, 2007; Odden *et al.*, 2011; Le *et al.*, 2015; Ding *et al.*, 2017; Wang *et al.*, 2017). However, relevant evidence from China is limited. To this end, our analyses of the influencing factors in the context of chronic disease management will fill in this literature gap.

## Conceptual framework

In order to address the prevalent chronic conditions, disease prevention is considered more significant than medical treatment, especially in terms of reducing hospitalizations and economic burden (Scott, 2008; Kim *et al.*, 2015). In the literature, evidence has been provided on the effectiveness of chronic disease management strategies in reducing health-care utilization and related medical costs of CHD in different countries (Clark *et al.*, 2005; Brady *et al.*, 2013; Hamar *et al.*, 2015). For instance, as reported by Hamar *et al.*, (2015), the Australian government established some chronic disease management programmes to reduce chronic disease morbidity and health-care utilization. It was reported that admissions, readmissions and length of stay in patients with heart diseases (CHD or heart failure) or diabetes were reduced by the intervention (Hamar *et al.*, 2015).

In line with the literature, we hypothesize that there will be fewer CHD casenesses and therefore fewer discharges, and the medical costs and bed days will be smaller after the implementation of a chronic disease management programme.

There are several ways in which chronic disease management is effective. First, a standardized chronic disease management programme can upgrade the process and quality of chronic disease health-care services (Clark *et al.*, 2005). Qualified health-care services are beneficial to the recovery of CHD patients. Medical costs and length of stay can be reduced. In addition, preventive care is usually included in the chronic disease management programme, which helps diminish the incidence of CHD and hospitalization is decreased as a result.

Second, coronary risk factors can be improved by disease management interventions. For example, total cholesterol was found to be lower in CHD patients after an intervention programme was conducted to train CHD patients on health management (Vale *et al.*, 2003). The implementation of chronic disease management interventions was reported to lower the blood pressure of patients with primary hypertension (Ling *et al.*, 2021).

Third, chronic disease management programmes advocate health behaviours. Patients tend to adopt more healthy behaviours when treated, such as physical exercise and healthy dietary patterns (Brady *et al.*, 2013; Hamar *et al.*, 2015). In addition, psychological health status is improved in the treated individuals (Brady *et al.*, 2013; Ling *et al.*, 2021). Both healthy behaviours and psychological health benefit individual physical health.

## Materials and methods

### Data source

A total of 6111 records were collected from a secondary Grade II public general hospital Y in central China, which has the largest number of clinical key disciplines (including cardiology) in the province. The hospital has almost 1000 employees and a capacity of 750 beds. Meanwhile, Y Hospital is leading an integrated health-care system involving 10 village hospitals, which serve population of more than 800 000 in total.

As one pilot unit of a county-scale chronic disease management programme, Y Hospital has been implementing the CDM programme based on its integrated health-care system since March 2019. In addition, with a China Chest Pain Center (national standard version, Level III) certification, Y Hospital provides the same qualified treatment (including drugs, devices, surgical methods and so on) for CHD as Grade III hospitals. Meanwhile, the technical methods of CHD treatment are diverse and comprehensive, including coronary angiography (CA) (non-stent) percutaneous coronary stent implantation for ischaemic heart disease (non-interventional) heart failure arrhythmias and severe arrhythmias (involving pacemakers and radiofrequency ablation) and so on.

Data originated from the medical files of CHD inpatients discharged from Y hospital between January 2017 and December 2020. The medical files were complete, which could retrospectively provide personal information, diagnosis results, treatment method and cost items records.

### Study sample

Every patient selected for our sample was judged to have CHD based on the diagnosis text by the doctor, combined with the ICD-10 codes (their diagnoses corresponding to I25.103 and I25.101) of their diagnosis. Among all 88 720 inpatient admissions in Y hospital over the 4-year period, 6111 (6.89%) were discharged with CHD as the primary diagnosis. Since there were 5515 (90.25% of 6111) first-time admissions, our sample was considered cross-sectional.

### Measures

#### Dependent variables

The changes in the direct medical cost and length of stay of CHD hospitalization were investigated. For those with medical insurance, the direct medical cost of hospitalization above deductible is partly reimbursed by medical insurance. The reimbursement rate varies across different types of medical insurance from 50% to 95% (Li *et al.*, 2018). The unreimbursed cost has to be paid by patients out of pocket. The direct medical cost data were obtained from the inpatient’s medical file. The total medical costs per discharge were the total bill charged by Y hospital, regardless of whether reimbursed or not, covering expenses on drugs, tests, echocardiography, angiography, surgery, imaging, ambulance and consultation. Provided in the file, as well, was the length of stay counted in days from the day of admission to the day of discharge.

Costs and prices were inflated to 2020 Chinese Yuan (RMB), using annual national inflation rates from 2017 to 2019. We estimated the medical cost per discharge rather than the total cost per each CHD patient per year. It is because, on the one hand, regular CHD medication and health monitoring usually happen in the outpatient department, which could also get reimbursed from medical insurance; on the other hand, the direct medical cost of CHD hospitalization that happens within the inpatient department is of main interest in this study, which is important to both CDM policy and medical insurance policy planning.

#### Main exposure

Since the implementation of chronic disease management programmes started from March 2019, a dummy variable *CDM* was constructed to indicate the existence of chronic disease management. *CDM* equals to 1 if it is after March 2019 (included), and 0 otherwise. Furthermore, with an assumption of log-linear time growth over month, centred time in month variable was constructed with a range from −26 to 21.

#### Covariates

A set of covariates was included, including individual age, gender, marital status, method of payment, the utilization of CA service and readmission plan. In addition, hypertension, diabetes, angina, myocardial infarction and hypercholesterolemia are the most common risk factors and frequent comorbidities of CHD (Tušek-Bunc and Petek, 2016; NHS, 2020). The diagnosis of these diseases when discharged was included as additional covariates considering their impact on CHD severity, treatment and medical cost. The corresponding ICD-10 codes of hypertension Stage I, II and III were I10.x03, I10.x04 and I10.x05 respectively. The ICD-10 codes of diabetes, angina, myocardial infarction and hypercholesterolemia were E11, I20, I21 and E78, respectively. Moreover, quarter fixed effects were included to control for seasonal heterogeneity (Hu *et al.*, 2015).

### Data analysis

We focused on the role of chronic disease management on CHD hospitalization, direct medical cost and length of stay. On the one hand, we conducted a univariate analysis to assess whether chronic disease management and individual attributes were statistically significant in affecting direct medical cost or length of stay. All the tests in the univariate and multivariate analyses were conducted on natural logarithm transformed data of direct medical cost or length of stay. On the other hand, to provide reliable evidence on the effect of chronic disease management on CHD hospitalization, we conducted a multivariate linear regression analysis, using the natural logarithm of total medical cost and length of stay as dependent variables, respectively. Additionally, we included centred time in month in the model, as well as an interaction term between *CDM* and time in month. Coefficients were exponentiated for better interpretation (Newson, 2003). In order to avoid the potential heteroskedasticity, robust standard errors were calculated. All statistical analyses were completed using StataSE 17. All statistical tests were two-sided, and *P* < 0.05 was considered statistically significant.

## Results

### Summary statistics

**Figure 1.**
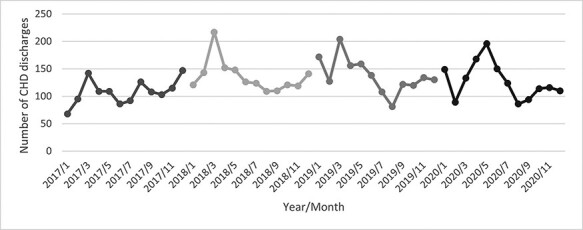
Monthly number of CHD discharges

**Figure 2.**
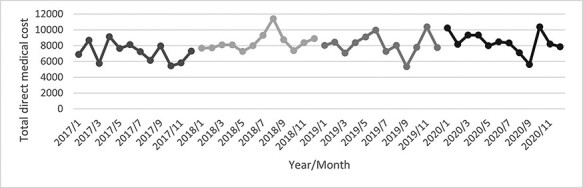
Monthly mean of total direct medical cost

**Figure 3.**
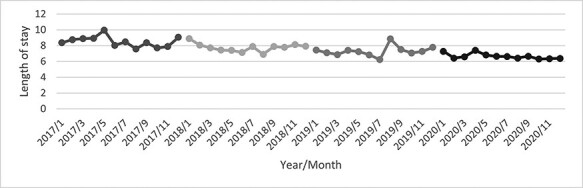
Monthly mean of length of stay

As shown in Figure 1, the number of monthly CHD discharges fluctuated. Despite an obvious increase in 2018, a decreasing trend in the monthly CHD discharges in 2019 and 2020 can be observed. Total direct medical cost fluctuated (Figure 2), whilst length of stay was declining over the years (Figure 3). Table 1 outlines the characteristics of all 6111 CHD inpatients in our working sample. The youngest CHD inpatient was 27 and the oldest aged 109. The mean age was 69.09 ± 12.07 years. The gender distribution was basically balanced. Most of the inpatients were married and had medical insurance. Within the 5947 inpatients with medical insurance, 4440 enrolled in the new rural cooperative medical scheme (NRCMS). The percentage of CA service utilization increased largely from 0.03% before CDM to 13.19% after CDM. There was a few (less than 1%) with readmission plan.

**Table 1.** Characteristics of sample (n = 6111)

	No. of inpatients discharges (%)		
Characteristic	Total	Before CDM	After CDM
Total	6111	3230	2881
Age			
Mean ± SD	69.09 ± 12.07	69.05 ± 11.92	69.13 ± 12.24
Gender			
Male	2982 (48.80)	1589 (49.20)	1393 (48.35)
Female	3129 (51.20)	1641 (50.80)	1488 (51.65)
Marital status			
Married	5378 (88.01)	2956 (91.52)	2872 (96.56)
Otherwise	733 (11.99)	274 (8.48)	459 (15.93)
Method of payment			
With insurance	5947 (97.32)	3109 (96.25)	2838 (98.51)
Self-supported	164 (2.68)	121 (3.75)	43 (1.49)
CA service			
Yes	381 (6.23)	1 (0.03)	380 (13.19)
No	5730 (93.77)	3229 (99.97)	2501 (86.81)
Readmission plan			
Yes	58 (0.95)	31 (0.96)	27 (0.94)
No	6053 (99.05)	3199 (99.04)	2854 (99.06)
Hypertension stage			
No	4308 (70.49)	2662 (82.45)	1646 (57.13)
I	39 (0.64)	9 (0.28)	30 (1.04)
II	410 (6.71)	129 (3.99)	281 (9.75)
III	1354 (22.16)	429 (13.28)	925 (32.11)
Diabetes			
Yes	735 (12.03)	324 (10.03)	411 (14.27)
No	5376 (87.97)	2906 (89.97)	2470 (85.73)
Angina			
Yes	621 (10.16)	173 (5.36)	448 (15.55)
No	5490 (89.84)	3057 (94.64)	2433 (84.45)
Myocardial infarction			
Yes	314 (5.14)	118 (3.65)	196 (6.80)
No	5797 (94.86)	3112 (96.35)	2685 (93.20)
Hypercholesterolemia			
Yes	157 (2.57)	58 (1.80)	99 (3.44)
No	5954 (97.43)	3172 (98.20)	2782 (96.56)

A total of 1803 (29.50%) CHD inpatients had hypertension. A few CHD inpatients were at hypertension Stage I. There were 410 inpatients at hypertension Stage II and 1354 inpatients at hypertension Stage III. Both, the proportion and severity of hypertension in CHD inpatients increased over time. There were about 12% of patients with diabetes, 10% with angina, 5% with myocardial infarction and 2.6% with hypercholesterolemia. The prevalence of these four comorbidities increased from before CDM to after CDM as well.

### Univariate analysis

Table 2 reports the univariate analysis results of total direct medical cost and length of stay. In all the CHD inpatients, the average total medical cost was 8419.73 Yuan and the average length of stay was 7.57 days. Despite a slightly higher direct medical cost after the implementation of chronic disease management, the length of stay was 1 day shorter when there was chronic disease management. Male inpatients paid significantly more for direct medical costs than female inpatients. Married inpatients had a significantly shorter length of stay in hospital. The direct medical cost and length of stay were both larger in inpatients with medical insurance compared with self-supported inpatients. CA service utilization caused a significant increase in medical costs but significantly fewer bed days in hospital. When there was a readmission plan, both medical cost and length of stay were lower. The direct medical costs of hypertension Stage-II and Stage-III inpatients were above the sample mean. Inpatients with hypertension Stage III had the highest total medical cost at 10 138.71 Yuan. Hypertension Stage-II inpatients incurred the shortest length of stay which was 6.44 days. ANOVA results indicated that the direct medical costs and length of stay between the four hypertension groups were significantly different at the 5% confidence level. A significantly higher medical cost was observed in CHD patients with diabetes, myocardial infarction or hypercholesterolemia. However, patients with angina or hypercholesterolemia spent fewer days in hospital compared with those without.

**Table 2.** Univariate analysis of CHD hospitalization (*n* = 6111)

	Medical cost		Length of stay	
	mean	p^a^	mean	p^a^
Total	8419.73		7.57	
CDM				
No	8346.79	**<0.01**	8.03	**<0.01**
Yes	8508.57		7.01	
Gender				
Male	9585.51	**<0.01**	7.70	0.54
Female	7308.16		7.45	
Marital status				
Married	8507.42	0.09	7.54	**0.01**
Otherwise	7762.06		7.85	
Method of payment				
With insurance	8523.15	**<0.01**	7.60	**<0.01**
Self-supported	4767.96		6.73	
CA service				
Yes	17 495.40	**<0.01**	5.09	**<0.01**
No	7888.94		7.72	
Readmission plan				
Yes	4827.51	**<0.01**	6.17	**<0.01**
No	8455.50		7.59	
Hypertension stage				
No	7809.27	**<0.01**	7.76	**<0.01**
I	6880.90		7.19	
II	9564.28		6.44	
III	10 138.71		7.29	
Diabetes				
Yes	10 922.25	**<0.01**	7.41	0.77
No	8062.37		7.59	
Angina				
Yes	9264.27	0.37	6.35	**<0.01**
No	8320.04		7.72	
Myocardial infarction				
Yes	28 940.76	**<0.01**	8.70	**<0.01**
No	7262.47		7.51	
Hypercholesterolemia				
Yes	10 453.34	**0.047**	6.10	**<0.01**
No	8363.96		7.61	

a Two-sample Student’s *t*-test or ANOVA for two groups or greater than two groups in the comparative analysis, respectively. Tests are conducted on natural logarithm transformed data. The bold values are considered statistically significant (*P* < 0.05).

### Regression results

Tables 3 and 4 report the multivariate linear regression results on direct medical cost and length of stay without or with additional time growth. Full adjustments were applied including age, age squared, gender, marital status, payment method, CA service utilization, readmission plan, comorbidities as well as quarter fixed effect.

**Table 3.** Multivariate linear regression analysis on medical cost and length of stay (*N* = 6111)

	Direct medical cost		Length of stay	
Variables	Ratio^a^	95% CI	Ratio^a^	95% CI
Main exposure				
CDM				
Yes vs No (ref)	0.7701*	(0.7344, 0.8075)	0.8848*	(0.8522, 0.9187)
Covariates				
Age	1.0186*	(1.0010, 1.0366)	1.0065	(0.9931, 1.0200)
Age squared	0.9998*	(0.9997, 1.0000)	1.0000	(0.9999, 1.0001)
Gender				
Male vs Female (ref)	1.1421*	(1.0959, 1.1903)	1.0208	(0.9868, 1.0560)
Marital status				
Married vs Otherwise (ref)	0.9775	(0.9172, 1.0418)	0.9886	(0.9384, 1.0415)
Method of payment				
Self-supported vs Insurance (ref)	0.5007*	(0.4114, 0.6093)	0.7944*	(0.6932, 0.9105)
CA service				
Yes vs No (ref)	1.9558*	(1.7691, 2.1622)	0.7219*	(0.6694, 0.7785)
Readmission plan				
Yes vs No (ref)	0.8446	(0.7111, 1.0031)	0.7158*	(0.5748, 0.8915)
Hypertension stage				
I vs No (ref)	0.9528	(0.7839, 1.1583)	0.9894	(0.7755, 1.2622)
II vs No (ref)	1.0285	(0.9425, 1.1223)	0.9248*	(0.8648, 0.9891)
III vs No (ref)	1.1307*	(1.0692, 1.1957)	1.0207	(0.9798, 1.0632)
Diabetes				
Yes vs No (ref)	1.1185*	(1.0435, 1.1989)	1.0365	(0.9861, 1.0895)
Angina				
Yes vs No (ref)	1.0722	(0.9932, 1.1575)	0.9119*	(0.8653, 0.9611)
Myocardial infarction				
Yes vs No (ref)	4.0220*	(3.6209, 4.4675)	1.3172*	(1.2252, 1.4160)
Hypercholesterolemia				
Yes vs No (ref)	1.0255	(0.8982, 1.1709)	0.8596*	(0.7781, 0.9497)
Constant^b^	3101.51*	(1693.76, 5679.30)	3.6746*	(2.3385, 5.7741)
Quarter FE	Yes		Yes	
*R*-squared	0.2135		0.0826	

a Ratios of outcome variable are reported (Newson, 2003).

b Constant indicates the baseline geometric mean of outcome variable.

* *P* < 0.05. FE: fixed effect; CI: confidence interval. Medical costs are at 2020 prices. Robust CI and standard errors are calculated.

**Table 4.** Multivariate linear regression analysis on medical cost and length of stay with additional time trend variable

	Direct medical cost		Length of stay	
Variables	Ratio^a^	95% CI	Ratio^a^	95% CI
Main exposure				
CDM				
Yes vs No (ref)	1.0183	(0.9362, 1.1076)	0.9676	(0.9046, 1.0351)
Time in month	0.9892*	(0.9854, 0.9930)	0.9907*	(0.9876, 0.9938)
Change in slope	0.9926*	(0.9860, 0.9992)	1.0126*	(1.0070, 1.0183)
Covariates				
Age	1.0173	(0.9998, 1.0351)	1.0062	(0.9928, 1.0197)
Age squared	0.9998*	(0.9997, 1.0000)	1.0000	(0.9999, 1.0001)
Gender				
Male vs Female (ref)	1.1422*	(1.0961, 1.1901)	1.0219	(0.9880, 1.0570)
Marital status				
Married vs Otherwise (ref)	0.9682	(0.9086, 1.0317)	0.9934	(0.9429, 1.0466)
Method of payment				
Self-supported vs Insurance (ref)	0.4852*	(0.3995, 0.5894)	0.7821*	(0.6834, 0.8950)
CA service				
Yes vs No (ref)	2.1932*	(1.9779, 2.4319)	0.7085*	(0.6540, 0.7676)
Readmission plan				
Yes vs No (ref)	0.8777	(0.7408, 1.0399)	0.7100*	(0.5706, 0.8835)
Hypertension stage				
I vs No (ref)	0.9954	(0.8184, 1.2107)	1.0137	(0.7971, 1.2893)
II vs No (ref)	1.0670	(0.9776, 1.1645)	0.9350	(0.8736, 1.0008)
III vs No (ref)	1.1713*	(1.1070, 1.2393)	1.0303	(0.9884, 1.0740)
Diabetes				
Yes vs No (ref)	1.1281*	(1.0525, 1.2090)	1.0326	(0.9823, 1.0855)
Angina				
Yes vs No (ref)	1.0875*	(1.0079, 1.1733)	0.9174*	(0.8706, 0.9667)
Myocardial infarction				
Yes vs No (ref)	4.0691*	(3.6664, 4.5159)	1.3358*	(1.2430, 1.4355)
Hypercholesterolemia				
Yes vs No (ref)	1.0355	(0.9089, 1.1797)	0.8620*	(0.7819, 0.9502)
Constant^b^	3646.2	(1998.22, 6653.31)	4.1681	(2.6489, 6.5584)
Quarter FE	Yes		Yes	
*R*-squared	0.2224		0.0881	

Notes. N = 6111.

a Ratios of outcome variable are reported (Newson, 2003).

b Constant indicates the baseline geometric mean of outcome variable.

* *P* < 0.05. FE: fixed effect; CI: confidence interval. Medical costs are at 2020 prices. Robust CI and standard errors are calculated.

It was indicated that the implementation of a county-scale chronic disease management programme significantly reduced direct medical costs and length of stay of CHD hospitalization. When there was chronic disease management, the total direct medical cost was 77.01% as high as that without chronic disease management, equivalent to a reduction of approximately 1956.12 Yuan at means. Meanwhile, the length of stay after chronic disease management was conducted was 88.48% as long, equivalent to a reduction of almost 1 day at means. With additional time growth included in the model, no significant change associated with chronic disease management was observed at the time point of policy implementation. However, chronic disease management was associated with a significantly faster-decreasing trend in direct medical costs. In terms of length of stay, chronic disease management reduced the length of stay in March 2019, while the time growth slope was increased.

Among the covariates, an inverted U-shape association between age and the medical cost was reported. Inpatients without medical insurance tended to spend significantly fewer medical costs and fewer days on hospitalization. Receiving CA services increased medical costs and decreased the length of stay simultaneously. It provided evidence on the effectiveness of CA services in CHD treatment. Compared with non-hypertensive inpatients, hypertension Stage III was a significant predictor of medical cost and hypertension Stage II significantly reduced the length of stay. Furthermore, the incidence of diabetes, angina or myocardial infarction was associated with significantly higher medical costs. In particular, myocardial infarction was associated with a 4-fold higher direct medical cost and a 30% longer stay in the hospital.

## Discussion

In this study, we estimated the change in the CHD hospitalization and its direct medical cost and length of stay after the implementation of a county-scale chronic disease management programme in a secondary public general hospital in China. During 2017–2020 in our studied county, CHD treatment in the outpatient department can get reimbursed from basic medical insurance without a deductible at a similar reimbursement rate to the inpatient department (with a deductible though). Thus, unnecessary admissions can be mostly avoided in our sample. Moreover, the yearly amount of reimbursement is restricted with a cap. Combined with the fact that most admissions in our sample are unique patients, it is reasonable to assume that splitting one admission into multiple admissions may rarely happen. Therefore, unnecessary, or multiple admissions are assumed inexistent.

From 2017 to 2020, the average direct medical cost of CHD treatment per admission at Y hospital was approximately 8419.73 Yuan and the average length of stay was 7.57 days. It is much cheaper than the cost incurred in Shanghai, China (about 15 642 Yuan in 2014) (Wang *et al.*, 2015), but more expensive than township hospitals in rural China (about 6250 Yuan in 2013/4) (Wang *et al.*, 2019). According to a study on hospitals in the same province as our hospital, the average hospitalization expenses of CHD patients were 9572 Yuan in a tertiary hospital and 5800 Yuan in a secondary hospital in 2018 (Yang *et al.*, 2018). In another province located in the central part of China, the hospitalization expenses of CHD patients from 2016 to 2018 were reported to be around 7700 Yuan (Wu *et al.*, 2021). A higher medical cost in our sample hospital than in other secondary hospitals is plausible because our sample hospital is a certified China Chest Pain Center. It can admit acute and critical CHD patients, such as those with CHD and heart failure. Compared with mostly mild admissions in other secondary hospitals, therapy is more complex and costly. In addition, these patients need more bed days for recovery.

Our findings provide evidence on the effectiveness of chronic disease management. According to regression results, chronic disease management programme significantly reduced CHD hospitalization medical cost by about 23% (1956.12 Yuan at means) and length of stay by about 11.5% (1 day at means). Meanwhile, the decreasing time trend in medical costs was enhanced by the programme. In the literature, decreased hospitalization, medical cost and bed days associated with chronic disease management were also observed (Adams *et al.*, 2007; Scott, 2008; Newman *et al.*, 2020). For example, a quality improvement programme aimed at children with asthma reduced asthma emergency department visits and hospitalizations. The medical cost was decreased as well (Woods *et al.*, 2012). In Australians with heart disease or diabetes, chronic disease management providing personalized health support lowered the odds of hospitalization utilization in terms of admissions, readmissions and bed days. In particular, the bed days were reduced by about 17.2% (Hamar *et al.*, 2015).

As illustrated by NHS, the most common risk factors of CHD include smoking, hypertension, high cholesterol, high lipoprotein(a), lack of exercise and diabetes (NHS, 2020). Accordingly, we were able to control the confounding effect of some comorbidities where hypertension had the highest prevalence. It was suggested that hypertension Stage III significantly increased the direct medical cost of CHD hospitalization compared with those without hypertension diagnosis. It was plausible since hypertension was associated with a higher risk and treatment cost of CHD (Loudon *et al.*, 2016; Kärner Köhler *et al.*, 2018; Murray *et al.*, 2018; Wang *et al.*, 2020). For example, hypertension incurred higher costs in acute coronary syndrome (ACS) treatment at a Swiss hospital (Bramkamp *et al.*, 2007). It is also supported by the abundant evidence on hypertension-attributable health-care costs (Kiiskinen *et al.*, 1998; Chodick *et al.*, 2010; Weaver *et al.*, 2015; Wang *et al.*, 2017).

There are several limitations to this study. First, our data were from one secondary public general hospital, which may not be representative of the whole country, hence the possibility of a sample bias that may slightly affect the accuracy of our results. Second, as mentioned earlier, there is a possibility of missing diagnoses of hypertension in our dataset. This may contribute to a sample bias in our distribution. Furthermore, even though we tried to include as many confounders as we can in the regression analysis, there were still some uncontrolled ones owing to limited data availability. Consequently, further investigation is needed to obtain a fuller picture of the medical costs of CHD treatment.

## Conclusion and policy implications

Aiming at evaluating the effectiveness of a county-scale chronic disease management programme in China, this study analysed discharges, hospitalization cost and length of stay of CHD inpatients in a secondary general hospital over a consecutive 4-year period. Our results suggest that the number of discharges decreased after the implementation of chronic disease management programme. Hospitalization medical cost was significantly reduced by 23% (1956.12 Yuan at means) and bed days were decreased by about 11.5% (almost 1 day at means). Moreover, chronic disease management was associated with a faster decreasing trend over time in the direct medical cost of CHD hospitalization. To conclude, chronic disease management is an effective way of relieving the medical and financial burden of hospitalization from both perspectives of providers and patients.

There are several implications of our study. First, chronic disease management and prevention should be substantially supported by health policy to help reduce the incidence risk of NCDs. Second, as one of the most important and prevalent risk factors of NCDs or cardiovascular diseases, hypertension management and prevention are calling for more attention from policymakers. Healthy lifestyle and dietary patterns should be advocated to decrease the risk of hypertension. Third, in order to minimize the waste of medical insurance funds, it is necessary to promote standardized treatment routes and cost-saving behaviours in health-care service providers.

## Data Availability

The data underlying this article cannot be shared publicly for the privacy of patients. The data will be shared on reasonable request to the corresponding author.

## Abbreviations

NCDs = non-communicable chronic diseases

CHD = coronary heart disease

CDM = chronic disease management

CA = coronary angiography

## Reflexivity statement

The authors include two females and two males and span multiple levels of seniority. The four authors are from various institutions and regions. While three authors are working in academia with different expertise including epidemiology, preventive medicine and public administration, the fourth is working in a hospital with abundant clinical and field experiences.
